# Bilateral erector spinae plane block on opioid-sparing effect in upper abdominal surgery: study protocol for a bi-center prospective randomized controlled trial

**DOI:** 10.1186/s13063-024-08612-w

**Published:** 2024-11-13

**Authors:** Changzhen Geng, Li Wang, Yaping Shi, Xinnan Shi, Hanyi Zhao, Ya Huang, Qiufang Ji, Yuanqiang Dai, Tao Xu

**Affiliations:** 1 Faculty of Anesthesiology, Changhai Hospital, Naval Medical University, Shanghai, China; 2 Department of Anesthesiology, Public Health Clinical Center of Shanghai, Shanghai, China

**Keywords:** Erector spinae plane block, Paravertebral block, Opioid-sparing effect, Upper abdominal surgery, Midline incision

## Abstract

**Background:**

Erector spinae plane block (ESPB) is a promising technique for effective analgesia. It is still uncertain if ESPB offers the same opioid-sparing effect as thoracic paravertebral block (PVB) in midline incision for upper abdominal surgery.

**Methods:**

The study is a prospective, bi-center, randomized, controlled, non-inferior trial. One hundred fifty-eight patients scheduled for upper abdominal surgery will be randomly assigned to receive bilateral ESPB or PVB before surgery. The primary outcome will be the equivalent cumulative analgesia dosage of sufentanil during the surgery, which is defined as the total dosage of sufentanil from anesthesia induction to tracheal extubation. The main secondary outcomes include postoperative complications and the quality of recovery-15 score at 24 h, 48 h, and 30 days after surgery.

**Discussion:**

This study will assess the opioid-sparing efficacy of ESPB and PVB, complications, and the quality of recovery of two blocks.

**Trial registration:**

ChiCTR2300073030 (https://www.chictr.org.cn/). Registered on 30 June 2023.

**Supplementary Information:**

The online version contains supplementary material available at 10.1186/s13063-024-08612-w.

## Administrative information

Note: the numbers in curly brackets in this protocol refer to SPIRIT checklist item numbers. The order of the items has been modified to group similar items (see http://www.equator-network.org/reporting-guidelines/spirit-2013-statement-defining-standard-protocol-items-for-clinical-trials/).

**Table:** 

Title {1}	Bilateral erector spinae plane block on opioid-sparing effect in upper abdominal surgery: study protocol for a bi-center prospective randomized controlled trial
Trial registration {2a and 2b}.	ChiCTR2300073030. https://www.chictr.org.cn/ [ClinicalTrials.gov] [Registered on 30 June 2023]
Protocol version {3}	Version 2.1 of 18–05-2023
Funding {4}	This work was supported by 234 Clinical Climb Project of Changhai Hospital affiliated to Naval Medical University (No. 2019YXK022).
Author details {5a}	Changzhen Geng, Faculty of Anesthesiology, Changhai Hospital, Naval Medical University, Shanghai, China & Department of Anesthesiology, Public Health Clinical Center of Shanghai, Shanghai, China; E-mail: changzhengeng@163.com Li Wang, Department of Anesthesiology, Public Health Clinical Center of Shanghai, Shanghai, China; E-mail: wanglimz@shphc.org.cn Yaping Shi, Faculty of Anesthesiology, Changhai Hospital, Naval Medical University, Shanghai, China; E-mail: shiyaping610733@sohu.com Xinnan Shi, Faculty of Anesthesiology, Changhai Hospital, Naval Medical University, Shanghai, China; E-mail: ntshixinnan@163.com Hanyi Zhao, Faculty of Anesthesiology, Changhai Hospital, Naval Medical University, Shanghai, China; E-mail: zhaohy522_cc@qq.com Ya Huang, Faculty of Anesthesiology, Changhai Hospital, Naval Medical University, Shanghai, China; E-mail: 1,527,542,179@qq.com Qiufang Ji, Faculty of Anesthesiology, Changhai Hospital, Naval Medical University, Shanghai, China; E-mail: 54,339,075@qq.com Yuanqiang Dai, Faculty of Anesthesiology, Changhai Hospital, Naval Medical University, Shanghai, China; E-mail: 413,650,671@qq.com Tao Xu, Faculty of Anesthesiology, Changhai Hospital, Naval Medical University, Shanghai, China; E-mail: xutaosci@163.com
Name and contact information for the trial sponsor {5b}	Investigator-initiated clinical trial; Tao Xu (Principal Investigator) xutaosci@163.com
Role of sponsor {5c}	This is an investigator-initiated clinical trial. Therefore, the funders played no role in the design of the study and collection, analysis, and interpretation of data and in writing the manuscript.

## Introduction

### Background and rationale {6a}

Upper abdominal surgery is one of the most common surgical procedures clinically [1–4]. Patients undergoing upper abdominal surgery have high pain intensity and opioid requirements, but opioid drugs present side effects including nausea, itching, sedation, hypotension to respiratory depression, and prolonged hospital stay [5–7]. Multimodal analgesia may reduce the consumption and adverse events of opioids after surgery. An important component of multimodal analgesia is peripheral nerve block [8–10].

Various nerve blocks combined with general anesthesia have recently been reported in upper abdominal surgery [11–14]. Paravertebral block (PVB) has been demonstrated as an effective analgesia for thoracic and abdominal surgery [15, 16], which has been confirmed to reduce perioperative opioid consumption by 45% [17]. However, despite its effectiveness, PVB is associated with potential complications such as intraoperative hypotension, pneumothorax, and neurovascular injury [18]. Erector spinae plane block (ESPB) emerged as an alternative method, which was described by Forero in 2016 [19]. Local anesthetic is injected into the fascial plane between erector spinae muscle (ESM) and the tip of the transverse process [20]. Local anesthetic spreads within this potential space over 3–6 vertebral levels in a cranio-caudal direction. Medial–lateral spread is usually confined to the boundaries of the erector spinae muscle, limited by its attachment to the angle of the ribs and the enveloping thoracolumbar fascia [21]. The mechanism of action originally proposed in the early descriptions of the ESP block was of local anesthetic spreading anteriorly from the plane of injection, through channels in the inter-transverse connective tissues, to the paravertebral space, where it could act on ventral rami and spinal nerve roots. Existing evidence demonstrates noninferiority of ESPB compared with PVB for postoperative analgesia, with fewer side effects for unilateral and bilateral thoracic surgery [22, 23].

The efficacy of ESPB compared with PVB in upper abdominal surgery has not been investigated thoroughly and requires future clinical trials [24, 25]. Therefore, we designed this prospective controlled non-inferior study to compare the opioid consumption and the quality of recovery between ESPB and PVB in midline incision upper abdominal surgery.

### Objectives {7}

This non-inferior study aims to compare the opioid consumption and quality of recovery between ESPB and PVB in midline incision upper abdominal surgery.

### Trial design {8}

**Fig. 1:**
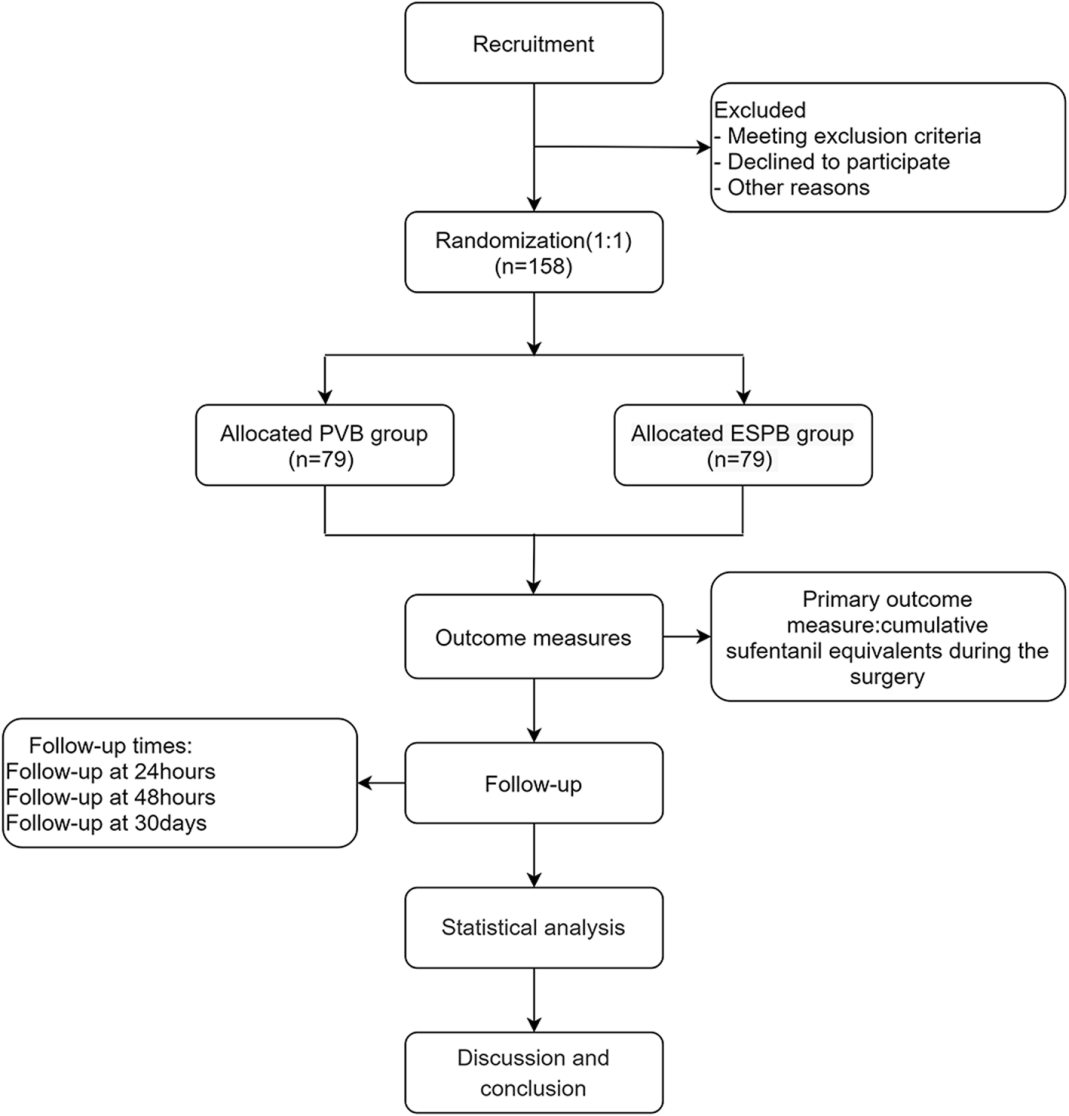
Flowchart of the trial design

This protocol also follows the updated CONSORT Statement (www.consortstatement.org), and the Standard Protocol Items: Recommendations for Interventional Trials (SPIRIT) Checklist (Appendix S1). A bi-center prospective randomized controlled and non-inferior trial will be conducted at the Changhai Hospital of Shanghai and the Public Health Clinical Center of Shanghai. The flowchart of this study is shown in Fig. 1.

## Methods: participants, interventions, and outcomes

### Study setting {9}

In this bi-center, prospective, randomized, non-inferiority trial, bilateral ESP block is compared to PVB on opioid-sparing effect in upper abdominal surgery. The patient allocation ratio is 1:1. Patients in the ESPB group and PVB group are respectively operated with an ultrasound-guided bilateral erector spinae muscle blockade and an ultrasound-guided bilateral paravertebral blockade.

### Eligibility criteria {10}

Inclusion criteria:

Patients scheduled for upper abdominal surgery between April 2023 and April 2024 will be included in this study:

1. American Society of Anesthesiologists (ASA) Physical Status classification: I–III
2. Patients aged 18–75 years
3. Median upper abdominal incision with an estimated operation time of 3 h ± 0.5 h
4. Participants provided written informed consent

Exclusion criteria:

Patients who refused inclusion or with any of the following conditions will not be included in this study:

1. Opioid abuse
2. Chronic pain and using analgesics before surgery
3. The therapy of corticosteroids before surgery
4. Motion sickness
5. Cognitive disorder
6. Contraindications for erector spinal block, paravertebral block (history of severe coagulopathy, infection, or allergy to local anesthetic)
7. 18.5 ≥ BMI ≥ 30 kg/m^2^
8. Severe heart disease and severe arrhythmia
9. Severe hepatic, renal disorders
10. Palliative surgery, emergent surgery, or reoperation

### Who will take informed consent? {26a}

All participants who are readily operated with midline incision upper abdominal surgery according to diagnoses by surgeons will be assessed for eligibility to participate in this study based on the inclusion criteria and exclusion criteria. A detailed explanation of the study process will be provided by a trained researcher to the participants in a private room, which mainly includes the objective, methods, privacy security, possible risks, and participant rights. All participants will be informed that they can withdraw from the study at any time with the quit reasons recorded. The written informed consent will be signed the day before the surgery and one of the copies will be kept by all participants.

### Additional consent provisions for collection and use of participant data and biological specimens {26b}

The clinical research coordinator authorized by the principal investigator will be involved in the organization and supervision during the whole study process. Patients will be identified by a numerical code and no personal information will be included in the case report form (CRF) and the database or will be included in the publication. No biological specimens of all participants will be collected and applied in this study.

## Interventions

### Explanation for the choice of comparators {6b}

This is a randomized, non-inferiority study comparing ESP block with PVB on opioid-sparing effect in upper abdominal surgery. The control group receives PVB block, which has been confirmed to reduce perioperative opioid consumption by 45% [17].

### Intervention description {11a}

#### PVB group

An ultrasound-guided bilateral paravertebral blockade will be performed by a senior anesthesiologist. With the patient lying in a prone position, the block will be performed with a SonoPlex®STIM nerve block tray (PAJUNK GmbH Medizintechnologie, 78,187 Geisingen, Germany). The imaginary horizontal line connecting T7 to the lower tip of the scapula will be used as a marker for recognition of T7. The needle will be inserted with an in-plane approach, passing through the costotransverse ligament into the paravertebral space under ultrasound guidance. Fifteen to 20 mL of 0.375% ropivacaine will be administered bilaterally.

#### ESPB group

The block will be performed with the same nerve block tray by a senior anesthesiologist. With the patient lying in a prone position, the needle will be inserted in-plane to contact the T7 transverse process. Correct needle tip position will be confirmed by a linear pattern of injectate spread lifting the erector spinae muscle off the transverse process. The same dosage of local anesthetics will be injected at this point.

Cold sensation will be assessed with ice 15–20 min after the end of injection to define the success of the block for both groups.

### Anesthesia induction and management

Immediately following completion of PVB or ESP blocks, patients will be returned to the supine position and general anesthesia will be induced with intravenous etomidate or propofol, sufentanil, midazolam or dexamethasone, and facilitated tracheal intubation with cisatracurium or rocuronium. Anesthesia induction is mainly based on the selection of experienced anesthesiologists, but sufentanil is a protocol choice. Mechanical ventilation will be then carried out. End-tidal carbon dioxide will be maintained at 35–45 mmHg during the surgery.

Anesthesia will be maintained using inhalation of sevoflurane 0.7–1.3 minimum alveolar concentration (MAC). Intermittent intravenous bolus of cisatracurium or rocuronium will be injected to maintain muscle relaxation during surgery. A bolus of 5–10 μg sufentanil will be supplemented if needed intraoperatively. Hypotension will be treated i.v. with an expedited infusion of phenylephrine (50 μg) or ephedrine (6 mg). Atropine (0.3–0.5 mg) was given if bradycardia (HR < 60 bpm) occurred.

### Criteria for discontinuing or modifying allocated interventions {11b}

Patients are advised that they are free to withdraw from the study at any time. The investigator may also withdraw the patient from the study at any time if perioperative management beyond the protocol of the trial is too much and hard to rescue. Any withdrawal of patients from the study will be documented in the final report. The decision to withdraw the research will be taken by the investigator.

If a nerve block is not implemented according to the protocol, it will be considered as modifying the allocated intervention. The patients will be excluded.

### Strategies to improve adherence to interventions {11c}

All blocks will be performed by a senior anesthesiologist, which will alleviate pain and relieve the anxiety of patients. A specialized follow-up group keeps close contact with subjects and surgeons to monitor postoperative rehabilitation. The sessions of treatments will be recorded in the CRF to assess the adherence of patients.

### Relevant concomitant care permitted or prohibited during the trial {11d}

Concomitant postoperative care such as the use of any additional medication or monitor of vital signs is permitted during the trial but will be registered. This study will not require a change in standard operating procedure (SOP) for participants performed with midline incision upper abdominal surgery. It is prohibited to perform additional nerve block or local anesthesia based on the intervention required by the plan.

### Provisions for post-trial care {30}

Intravenous patient-controlled analgesia of sufentanil (1.5 ~ 2 μg/kg) and ketorolac (150 ~ 180 mg) (loading dose at 2 ml, background dose at 2 ml/h and lockout interval of 15 min) was initiated after tracheal extubation. Participants were observed in the post-anesthesia care unit for > 30 min before being transferred to a ward or intensive care unit. Intravenous parecoxib (40 mg) will be available at the patient’s request as additional analgesia when pain control is insufficient.

### Outcomes {12}

#### Primary outcome measures

The primary outcome of this study will be the equivalent cumulative analgesia dosage of sufentanil during the surgery, which will be defined as the total dosage of sufentanil from anesthesia induction to tracheal extubation.

#### Secondary outcomes measures

The secondary outcomes are the quality of recovery-15 (QOR-15) score at 24 h, 48 h, and 30 days after surgery. The following will also be recorded: relevant complications of PVB and ESPB such as infection and pneumothorax, adverse effects of opioid use including nausea or vomiting to 48 h, consumption of vasopressors, surgical complications (infection, bleeding, anastomotic leak, stress ulcer), time to drain removal, time to ingestion and ambulation, time to hospital discharge.

### Participant timeline {13}

The participant timeline is described in Table 1.

**Table 1:** Participant timeline

	**Baseline**	**Interventions**	**Follow-up**		
**Timepoint (day 0 = day of surgery)**	**Day -1**	**Day 0**	**Day 1**	**Day 2**	**Day 30**
**Participants**					
Eligibility screen	√				
Demography	√				
Informed consent	√				
Health history	√				
Randomization		√			
**Interventions**					
ESPB group		√			
PVB group		√			
**Outcomes**					
Intraoperative sufentanil equivalents		√			
**QoR-15**			√	√	√
Complications of nerve block					
Infection		√	√	√	√
Pneumothorax		√	√	√	√
Adverse effects of opioid use		√	√	√	√
Consumption of vasopressors		√			
Surgical complications		√	√	√	√
Time to drain removal			√	√	√
Time to ingestion and ambulation			√	√	√
Hospital discharge			√	√	√

*ESPB group* erector spinae plane block group, *PVB group* thoracic paravertebral block group, *QoR-15* the quality of recovery-15 score

### Sample size {14}

This study uses a non-inferiority trial design, and the primary outcome is equivalent cumulative analgesia dosage of sufentanil during the surgery. The sample size is calculated based on the difference between the two groups of cumulative sufentanil equivalents during the surgery. The equivalent cumulative analgesia dosage of sufentanil of the patients in the PVB group is estimated to be 40 μg based on clinical data from our previous study [17]. We defined an acceptable non-inferiority margin as 10 μg. We calculated that each group should have 72 participants in order to achieve 80% power to distinguish a cumulative sufentanil consumption after erector spinae block that is 10% higher than after paravertebral block, at a significance level of 0.05. This calculation is based on the standard deviations in consumption, which were found to be 12 μg after erector spinae block and 12 μg after paravertebral block. Given the rate of a dropout and block failure was 10%, we enrolled 79 participants per group. One hundred eighteen patients were enrolled at the Changhai Hospital of Shanghai, and 44 subjects were included at the Public Health Clinical Center of Shanghai according to surgeon procedures annually.

### Recruitment {15}

Patient screening is conducted 1 day before the surgery based on preoperative arrangements; then, eligible patients will be enrolled according to the admission criteria. After sufficient information is provided and informed consent is signed, recruitment can be carried out.

## Assignment of interventions: allocation

### Sequence generation {16a}

Participants were randomly allocated to either the PVB group or ESPB group in a 1:1 ratio via a computer-generated list.

### Concealment mechanism {16b}

The allocation of each patient will be concealed in an opaque envelope, which will be only opened on the day of surgery by the anesthesiologist performing the blocks.

### Implementation {16c}

A specialized researcher who is responsible for statistical analysis will generate the allocation via the SPSS software (V.25.0; SPSS) or R. (version 4.2.3).

## Assignment of interventions: blinding

### Who will be blinded {17a}

The trunk blocks will be performed by an experienced anesthesiologist. The investigator is not involved in outcome assessment or further patient management. All other personnel involved in intraoperative and postoperative care of the patients (anesthesiologists, surgeons, and nursing staff), as well as assessors of study outcomes, are blinded to group allocation.

### Procedure for unblinding if needed {17b}

If serious complications occur, they will be recorded during the trial. The emergency will be reported to the trial steering committee who considers that may be related to the intervention of the trail and requires emergent unblinding; then, unblinding can be carried out.

## Data collection and management

### Plans for assessment and collection of outcomes {18a}

The preoperative characteristics of participants will be retrieved from electronic patient records. The data collected for the trial will be registered with a standardized case report form (CRF). Then, a specialized researcher entered data from CRF in Excel (Microsoft Excel version 2016) for statistical analysis.

### Plans to promote participant retention and complete follow-up {18b}

Plans are made to promote participant retention and complete follow-up, including satisfying patients by providing extensive information about the study and popular science of midline incision abdominal surgery. Moreover, the researchers will contact patients 5 days and 15 days after hospital discharge to improve the follow-up at 30 days.

### Data management {19}

The CRF will be documented by the doctors participating in the study, and in each selected case, it must be completed. After the completed CRF is reviewed by the clinical supervisor, the original form will be handed over to the data manager for data entry and management. The trial will set up a database using Excel 2016 to manage the study data. The doctor who is responsible for statistical analysis will have access to the dataset for the final analysis of trial outcomes.

### Confidentiality {27}

Research data will be stored using a study identification code for each participant. The key to the identification code list will only be available to the research team during the study and will be documented and safeguarded by the principal investigator according to research guidelines after completion of the study. No patient identification details will be reported in publications.

### Plans for collection, laboratory evaluation, and storage of biological specimens for genetic or molecular analysis in this trial/future use {33}

In this trial, no biological specimens will be collected and applied.

## Statistical methods

### Statistical methods for primary and secondary outcomes {20a}

All statistical analyses will follow the principle of intention-to-treat (ITT). All patients who underwent randomization will be included in the final analysis. A two-sided test at a significance level of 95% is deemed as statistically significant. For the description of baseline features, the mean with the standard deviation or median with interquartile range (IQR) will be used for continuous data, based on whether it conforms to a normal distribution. The primary outcome was analyzed according to the non-inferiority approach. The non-inferiority hypothesis for the primary outcome was tested using the two-sided *t*-test under a significance level of 5%. Continuous variables were analyzed using the *t*-test or Mann–Whitney *U* test as appropriate. Numeric data were reported as mean (standard deviation (SD)) or median (interquartile range (IQR)). Categorical variables were analyzed using the *χ*^2^ test or Fisher’s exact test. Bonferroni correction was used for multiple comparisons. The mean standard deviation (MSD) was utilized to assess inter-group disparities. If the MSD ≥ 0.2, it was considered that there were significant differences between groups. For parameters with an MSD ≥ 0.2, inverse probability weighting matching was performed for sensitivity analysis. Data analyses were performed using SPSS software (V.25.0; SPSS). For all analyses, *p* < 0.05 was taken to indicate significance.

### Interim analyses {21b}

The interim analysis will be conducted when half of the sample size is completed, to observe the trend of outcomes and double-check the trial process.

### Methods for additional analyses (e.g., subgroup analyses) {20b}

The subgroup situations for different types of surgeries will be presented in the descriptive analysis. However, further post hoc subgroup analyses may be limited by sample size and cannot be known in advance. Additionally, considering the limitation that subgroup analyses should not be overinterpreted, no specific prospective subgroup analysis plan was added to this protocol. Nevertheless, based on the statistical results from the descriptive analysis, appropriate discussions on subgroup will be conducted in the discussion section if necessary.

### Methods in analysis to handle protocol non-adherence and any statistical methods to handle missing data {20c}

If the missing data is less than 10%, analyses will be performed according to the principle of intention-to-treatment. If the missing data is greater than 10%, data imputation will be adopted for analyses.

### Plans to give access to the full protocol, participant-level data, and statistical code {31c}

The full protocol, participant-level dataset, and statistical code will be not publicly available but can be made available from corresponding author on reasonable request.

## Oversight and monitoring

### Composition of the coordinating center and trial steering committee {5d}

Two anesthesiologists from the Department of Anesthesiology at Changhai Hospital form the coordinating center. This group will meet weekly and is responsible for preparation of the trial, communication with the ethics committee and sponsors, assistance in the implementation of the trial, and coordinating the work of the researchers at the center. The trial steering committee is composed of one expert from the faculty of anesthesiology, one expert from the department of pharmacy, and one statistical expert. The group is responsible for overseeing the conduct of clinical trial, reviewing progress of study, and agreeing changes to the protocol and/or investigator brochure to facilitate the smooth running of the study. Recruitment of patients and liaising with the principal investigator are their responsibility too.

### Patient public involvement and public involvement group

The clinical trial requires active patient engagement, which is facilitated by ongoing communication between the coordinating trial group and potential participants. This approach is designed to ensure that patients can comprehensively understand the trial and are able to give informed consent. Transparent communication is an important method to strengthen the interest of participants on this trial and the trust of participants with the group, which can help build a harmonious and positive atmosphere.

### Composition of the data monitoring committee, its role and reporting structure {21a}

The data monitoring committee (DMC) is composed by specialized staff of Changhai Hospital responsible for the study safety in Changhai Hospital and Public Health Clinical Center of Shanghai. Any AEs happened will be recorded on a CRF and reported to the DMC. The data monitoring committee is responsible for monitoring the progress of the trial, reviewing trial data, evaluating the ethical conduct of the trial, and providing recommendations and opinions. The principal investigators (PI) will provide progress reports, safety reports, efficacy reports, and ethical reports during the mid-term of the trial. The monitoring committee will approve the relevant reports based on their content and on-site investigations and formulate recommendations and opinions.

### Adverse event reporting and harms {22}

The adverse events (AEs) of PVB and ESPB include inadvertent vascular puncture (3.8% to 6.8%), hypotension (4.0% to 4.6%), hematoma (2.4%), pain at the site of skin puncture (1.3%), signs of epidural or intrathecal spread (1.0%), pleural puncture (0.8% to 1.1%), local anesthetic toxicity, and pneumothorax (0.5%). Adverse events should be recorded on a CRF and marked in detail by the investigator, signed, and dated. The principal investigator must take action to protect the safety of subjects once a serious adverse event appears. The investigator should also report to the ethics committee and the data monitoring committee of Changhai Hospital. To reduce the occurrence of AEs, the process of intervention will strictly abide by aseptic principles and clinical practices and the nerve block will be operated by a senior clinician under the guidance of ultrasound.

### Frequency and plans for auditing trial conduct {23}

The project management group is composed by the principal investigator and the director of anesthesiology department in Changhai Hospital and Public Health Clinical Center of Shanghai. They will meet monthly to review progress on the study. Additionally, the trial steering group and the independent data monitoring and ethics committee play critical roles in ongoing monitoring and review. These groups will be responsible for overseeing trial integrity, making decision related to the trial conduct and protecting the safety and welfare of participants. A start-up meeting will be hold on the beginning of the trial by these groups, followed by planned review meetings every three months during the trial for auditing trial conduct.

### Plans for communicating important protocol amendments to relevant parties (e.g., trial participants, ethical committees) {25}

Because this is a study initiated by the principal investigator (PI), all protocol modifications (e.g., changes to eligibility criteria, outcomes, and analyses) will be communicated with the PI. Then, the PI will notify the coordinating centers, and a copy of the revised protocol will be sent to the PI to add to the Investigator Site File. Any deviations from the protocol will be fully documented using a breach report form. And online trail registries in ClinicalTrials.gov will be updated. Promptly communicating, recording, and updating information can ensure transparency, accuracy, and compliance of the research process.

### Dissemination plans {31a}

The results of this research will be attempted to be published in international journals.

## Discussion

Despite the rapid development of new technologies such as minimally invasive, endoscopy and intervention, traditional midline incision upper abdominal surgery still involves a large proportion of elective and emergency surgical cases, especially for necessary lymph node dissection and removal of tumors rich in blood vessels [26–28]. Patients undergoing major upper abdominal surgery are confronted with severe pain and have a high analgesia requirement. Poorly controlled postoperative pain is associated with increased morbidity, negatively affects quality of life and functional recovery, and is a risk factor for persistent pain or opioid abuse [29–31].

Opioid-related side effects and the opioid abuse epidemic emphasize the need for alternative, opioid-sparing analgesic strategies, including neuraxial (epidural/intrathecal) techniques, truncal nerve blocks, and local infusions [32]. Epidural analgesia (EA) is known to provide effective analgesia due to its superior efficacy [33]. However, EA is reported with a high failure rate (13% ~ 32%) and a risk of major complications [34, 35]. Compared with EA, PVB has been proven to be one of the most valid regional anesthesia techniques for practical postoperative analgesia [36]. However, this is also a particularly challenging technique because of the anatomic proximity of the pleura and central neuraxial system [37]. Recently bilateral ESP block seems like a suitable alternative to PVB because both target the thoracoabdominal nerves important for abdominal analgesia [38]. The simple application and safety of ESP block have increased its acceptability [5, 39, 40]. Thus, ESP block has been studied in a small number of randomized trials involving various types of abdominal surgery [41–44], but these papers mainly concentrated on lower abdominal surgery and surgery with lower analgesia requirements. Limited research exists on analgesia effects and opioid-sparing using ESP block technique for patients undergoing midline incision upper abdominal surgery. Therefore, we expect that this study will provide evidence for the analgesia of upper abdominal surgery in the view of the opioid-sparing. We also believe that this study will benefit patients by alleviating their pain to facilitate their rehabilitation.

In this trial, we hypothesize that erector spinae plane block will provide non-inferior analgesic effectiveness, and fewer postoperative complications when compared with a paravertebral block, which had been proven with excellent analgesic effects in upper abdominal surgery in our previous study [17]. As anesthesiologists, the safety and necessity of implementing nerve blocks must be carefully assessed by analyzing risks and benefits. The ultrasound guidance in-plane technique of PVB is reported of potential risks such as pneumothorax and vascular puncture [45]. Therefore, we designed this study to evaluate whether the ESPB with operation simplicity could be an appropriate alternative, especially in the opioid-sparing for upper abdominal surgery. An acceptable non-inferiority margin of 10 is defined in our trial. To our knowledge, this is the first trial to assess whether ESPB provides sufficient opioid-sparing effect in upper abdominal surgery. The study results will provide more convincing evidence-based information of ESPB and promote its application in upper abdominal surgery.

## Trial status

The protocol version number is 2.1 of 18–05-2023. The protocol was made on 18 May 2023. The research began in July 2023, and a few patients participated in the trial. Inclusion is expected to be completed in June 2024 according to the surgeon’s procedure volume.

## Supplementary Information

Supplementary Material 1.

## Data Availability

The dataset used and/or analyzed after completion of this research will be provided by the corresponding author upon reasonable request.

## Abbreviations

ESPB: Erector spinae plane block
PVB: Paravertebral block
ESM: Erector spinae muscle
SPIRIT: Standard Protocol Items: Recommendations for Interventional Trials
CONSORT: Consolidated Standards of Reporting Trials
CRF: Case report form
ASA: American Society of Anesthesiologists
MAC: Minimum alveolar concentration
SOP: Standard operating procedure
AEs: Adverse events
ITT: Intention-to-treat
SD: Standard deviation
IQR: Interquartile range
QOR-15: Quality of recovery-15
MSD: Mean standard deviation
PI: Principal investigator
EA: Epidural analgesia

## References

[1] Latenstein C, Hannink G, Bilt JVD, Donkervoort S, Eijsbouts Q, Heisterkamp J, et al. A clinical decision tool for selection of patients with symptomatic cholelithiasis for cholecystectomy based on reduction of pain and a pain-free state following surgery. JAMA Surg. 2021;156(10):e213706. 34379080 10.1001/jamasurg.2021.3706 PMC8358816

[2] Dagyaran I, Olesen CM, Brix LD. Patient-experienced quality during postoperative pain management - a phenomenological-hermeneutic study. J Perianesth Nurs. 2022;37(2):253–9. 34774420 10.1016/j.jopan.2021.09.007

[3] O’Neill A, Lirk P. Multimodal analgesia. Anesthesiol Clin. 2022;40(3):455–68. 36049874 10.1016/j.anclin.2022.04.002

[4] Villa G, Lanini I, Amass T, Bocciero V, Grotto RL. Effects of psychological interventions on anxiety and pain in patients undergoing major elective abdominal surgery: a systematic review. Perioper Med. 2020;9(1):38. 10.1186/s13741-020-00169-x PMC7722323 33292558

[5] Hemmerling TM. Pain management in abdominal surgery. Langenbecks Arch Surg. 2018;403(7):791–803. 30284029 10.1007/s00423-018-1705-y

[6] Fawcett WJ, Baldini G. Optimal analgesia during major open and laparoscopic abdominal surgery. Anesthesiol Clin. 2015;33(1):65–78. 25701929 10.1016/j.anclin.2014.11.005

[7] Cron DC, Englesbe MJ, Bolton CJ, Joseph MT, Carrier KL, Moser SE, et al. Preoperative opioid use is independently associated with increased costs and worse outcomes after major abdominal surgery. Ann Surg. 2017;265(4):695–701. 27429021 10.1097/SLA.0000000000001901

[8] Sanderson BJ, Doane MA. Transversus abdominis plane catheters for analgesia following abdominal surgery in adults. Reg Anesth Pain Med. 2018;43(1):5–13. 29099414 10.1097/AAP.0000000000000681

[9] Pirie K, Doane MA, Riedel B, Myles PS. Analgesia for major laparoscopic abdominal surgery: a randomised feasibility trial using intrathecal morphine. Anaesthesia. 2022;77(4):428–37. 35038165 10.1111/anae.15651

[10] Thomas GJ, Bauman JC, Bergeron S, Wasvary HJ, Ziegler MA. Perioperative lidocaine infusion reduces opioid use in enhanced recovery after surgery patients undergoing laparoscopic colectomy. Am Surg. 2023;89(11):4806–10. 36318225 10.1177/00031348221135785

[11] Desai N, El-Boghdadly K, Albrecht E. Epidural vs. Transversus abdominis plane block for abdominal surgery - a systematic review, meta-analysis and trial sequential analysis. Anaesthesia. 2021;76(1):101–17. 32385856 10.1111/anae.15068

[12] Abdel-Ghaffar HS, Askar FGE, Mohamed HH, Ibrahim NM, Abdel-Wahab AH, Abbas MS. Analgesic and respiratory effects of two doses of morphine as an adjunct to bupivacaine in ultrasound-guided transversus abdominis plane block in upper abdominal surgery. Pain Physician. 2019;22(5):509–17. 31561652

[13] Altiparmak B, Toker MK, Uysal AI, Kusçu Y, Demirbilek SG. Ultrasound-guided erector spinae plane block versus oblique subcostal transversus abdominis plane block for postoperative analgesia of adult patients undergoing laparoscopic cholecystectomy: randomized, controlled trial. J Clin Anesth. 2019;57:31–6. 30851501 10.1016/j.jclinane.2019.03.012

[14] Zhu Q, Li L, Yang Z, Shen J, Zhu R, Wen Y, et al. Ultrasound guided continuous quadratus lumborum block hastened recovery in patients undergoing open liver resection: a randomized controlled, open-label trial. BMC Anesthesiol. 2019;19:23. 30777027 10.1186/s12871-019-0692-z PMC6380018

[15] Schreiber KL, Chelly JE, Lang RS, Abuelkasem E, Geller DA, Marsh JW, et al. Epidural versus paravertebral nerve block for postoperative analgesia in patients undergoing open liver resection: a randomized clinical trial. Reg Anesth Pain Med. 2016;41(4):460–8. 27281726 10.1097/AAP.0000000000000422

[16] Alimian M, Imani F, Rahimzadeh P, Faiz SHR, Bahari-Sejahrood L, C Hertling A. Adding dexmedetomidine to bupivacaine in ultrasound-guided thoracic paravertebral block for pain management after upper abdominal surgery: a double-blind randomized controlled trial. Anesthesiol Pain Med. 2021;11(6):e120787-e. 10.5812/aapm.120787 PMC8908442 35291399

[17] Han Y, Dai Y, Shi Y, Zhang X, Xia B, Ji Q, et al. Ultrasound-guided paravertebral blockade reduced perioperative opioids requirement in pancreatic resection: a randomized controlled trial. Front Surg. 2022;9:903441. 36111230 10.3389/fsurg.2022.903441 PMC9468231

[18] Ardon AE, Lee J, Franco CD, Riutort KT, Greengrass RA. Paravertebral block: anatomy and relevant safety issues. Korean J Anesthesiol. 2020;73(5):394–400. 32172551 10.4097/kja.20065 PMC7533185

[19] Forero M, Adhikary SD, Lopez H, Tsui C, Chin KJ. The erector spinae plane block: a novel analgesic technique in thoracic neuropathic pain. Reg Anesth Pain Med. 2016;41(5):621–7. 27501016 10.1097/AAP.0000000000000451

[20] Cui Y, Wang Y, Yang J, Ran L, Zhang Q, Huang Q, et al. The effect of single-shot erector spinae plane block (ESPB) on opioid consumption for various surgeries: a meta-analysis of randomized controlled trials. J Pain Res. 2022;15:683–99. 35281481 10.2147/JPR.S346809 PMC8910495

[21] Chin KJ, El-Boghdadly K. Mechanisms of action of the erector spinae plane (ESP) block: a narrative review. Can J Anaesth. 2021;68(3):387–408. 33403545 10.1007/s12630-020-01875-2

[22] Aksu C, Sen MC, Akay MA, Baydemir C, Gurkan Y. Erector spinae plane block vs quadratus lumborum block for pediatric lower abdominal surgery: a double blinded, prospective, and randomized trial. J Clin Anesth. 2019;57:24–8. 30851499 10.1016/j.jclinane.2019.03.006

[23] Chen N, Qiao Q, Chen R, Xu Q, Zhang Y, Tian Y. The effect of ultrasound-guided intercostal nerve block, single-injection erector spinae plane block and multiple-injection paravertebral block on postoperative analgesia in thoracoscopic surgery: a randomized, double-blinded, clinical trial. J Clin Anesth. 2020;59:106–11. 31330457 10.1016/j.jclinane.2019.07.002

[24] Koushik SS, Bui A, Slinchenkova K, Badwal A, Lee C, Noss BO, et al. Analgesic techniques for rib fractures-a comprehensive review article. Curr Pain Headache Rep. 2023. 10.1007/s11916-023-01172-9. 37747621 10.1007/s11916-023-01172-9

[25] Viderman D, Aubakirova M, Abdildin Y. Erector spinae plane block in abdominal surgery: a meta-analysis. Front Med. 2022;9:812531. 10.3389/fmed.2022.812531 PMC8904394 35280917

[26] Seiler CM, Deckert A, Diener MK, Knaebel H-P, Weigand MA, Victor N, et al. Midline versus transverse incision in major abdominal surgery: a randomized, double-blind equivalence trial (povati: Isrctn60734227). Ann Surg. 2009;249(6):913–20. 19474689 10.1097/SLA.0b013e3181a77c92

[27] Hollinsky C. Individualized or standard approach to the abdomen. Currently available data. Chirurg. 2016;87(9):731–6. 27356925 10.1007/s00104-016-0221-2

[28] Cauchy F, Fuks D, Nomi T, Schwarz L, Barbier L, Dokmak S, et al. Risk factors and consequences of conversion in laparoscopic major liver resection. Br J Surg. 2015;102(7):785–95. 25846843 10.1002/bjs.9806

[29] Dunkman WJ, Manning MW. Enhanced recovery after surgery and multimodal strategies for analgesia. Surg Clin North Am. 2018;98(6):1171–84. 30390850 10.1016/j.suc.2018.07.005

[30] Baig MK, Zmora O, Derdemezi J, Weiss EG, Nogueras JJ, Wexner SD. Use of the ON-Q pain management system is associated with decreased postoperative analgesic requirement: double blind randomized placebo pilot study. J Am Coll Surg. 2006;202(2):297–305. 16427556 10.1016/j.jamcollsurg.2005.10.022

[31] Pirie K, Traer E, Finniss D, Myles PS, Riedel B. Current approaches to acute postoperative pain management after major abdominal surgery: a narrative review and future directions. Br J Anaesth. 2022;129(3):378–93. 35803751 10.1016/j.bja.2022.05.029

[32] Hμghes MJ, Ventham NT, McNally S, Harrison E, Wigmore S. Analgesia after open abdominal surgery in the setting of enhanced recovery surgery a systematic review and meta-analysis. Jama Surg. 2014;149(12):1224–30. 25317633 10.1001/jamasurg.2014.210

[33] Guay J, Nishimori M, Kopp SL. Epidural local anesthetics versus opioid-based analgesic regimens for postoperative gastrointestinal paralysis, vomiting, and pain after abdominal surgery: a cochrane review. Anesth Analg. 2016;123(6):1591–602. 27870743 10.1213/ANE.0000000000001628

[34] McLeod GA, Davies HTO, Munnoch N, Bannister J, Macrae W. Postoperative pain relief using thoracic epidural analgesia: outstanding success and disappointing failures. Anaesthesia. 2001;56(1):75–81. 11167441 10.1046/j.1365-2044.2001.01763-7.x

[35] Unic-Stojanovic D, Babic S, Jovic M. Benefits, risks and complications of perioperative use of epidural anesthesia. Med Arch (Sarajevo, Bosnia and Herzegovina). 2012;66(5):340–3. 10.5455/medarh.2012.66.340-343 23097975

[36] Richardson J, Lönnqvist PA, Naja Z. Bilateral thoracic paravertebral block: potential and practice. Br J Anaesth. 2011;106(2):164–71. 21233114 10.1093/bja/aeq378

[37] Slinchenkova K, Lee K, Choudhury S, Sundarapandiyan D, Gritsenko K. A review of the paravertebral block: benefits and complications. Curr Pain Headache Rep. 2023;27(8):203–8. 37294514 10.1007/s11916-023-01118-1

[38] Portela DA, Castro D, Romano M, Gallastegui A, Garcia-Pereira F, Otero PE. Ultrasound-guided erector spinae plane block in canine cadavers: relevant anatomy and injectate distribution. Vet Anaesth Analg. 2020;47(2):229–37. 31980367 10.1016/j.vaa.2019.10.005

[39] Zhao H, Xin L, Feng Y. The effect of preoperative erector spinae plane vs. paravertebral blocks on patient-controlled oxycodone consumption after video-assisted thoracic surgery: a prospective randomized, blinded, non-inferiority study. J Clin Anesth. 2020;62:109737. 32092617 10.1016/j.jclinane.2020.109737

[40] Tulgar S, Kapakli MS, Senturk O, Selvi O, Serifsoy TE, Ozer Z. Evaluation of ultrasound-guided erector spinae plane block for postoperative analgesia in laparoscopic cholecystectomy: a prospective, randomized, controlled clinical trial. J Clin Anesth. 2018;49:101–6. 29913392 10.1016/j.jclinane.2018.06.019

[41] Park YJ, Chu S, Yu E, Joo JD. Comparison of the efficacy of erector spinae plane block according to the difference in bupivacaine concentrations for analgesia after laparoscopic cholecystectomy: a retrospective study. J Yeungnam Med Sci. 2023;40(2):172–8. 36137572 10.12701/jyms.2022.00500 PMC10076922

[42] Hamed MA, Goda AS, Basiony MM, Fargaly OS, Abdelhady MA. Erector spinae plane block for postoperative analgesia in patients undergoing total abdominal hysterectomy: a randomized controlled study original study. J Pain Res. 2019;12:1393–8. 31118757 10.2147/JPR.S196501 PMC6503185

[43] Kamel AAF, Amin OAI, Ibrahem MAM. Bilateral ultrasound-guided erector spinae plane block versus transversus abdominis plane block on postoperative analgesia after total abdominal hysterectomy. Pain Physician. 2020;23(4):375–82. 32709172

[44] Dost B, Kaya C, Ozdemir E, Ustun YB, Koksal E, Bilgin S, et al. Ultrasound-guided erector spinae plane block for postoperative analgesia in patients undergoing open radical prostatectomy: a randomized, placebo-controlled trial. J Clin Anesth. 2021;72:110277. 33838536 10.1016/j.jclinane.2021.110277

[45] D’Ercole F, Arora H, Kumar PA. Paravertebral block for thoracic surgery. J Cardiothorac Vasc Anesth. 2018;32(2):915–27. 29169795 10.1053/j.jvca.2017.10.003

